# Clinical T1/2 renal cell carcinoma: multiparametric dynamic contrast-enhanced MRI features-based model for the prediction of individual adverse pathology

**DOI:** 10.1186/s12957-024-03431-4

**Published:** 2024-06-01

**Authors:** Keruo Wang, Baoyin Guo, Zhili Yao, Gang Li

**Affiliations:** 1 Department of Urology, Tianjin Institute of Urology, The Second Hospital of Tianjin Medical University, Tianjin, 300211 China; 2 Department of Urology, Tianjin Baodi Hospital, Baodi Clinical College of Tianjin Medical University, Tianjin, 301800 China

**Keywords:** Renal cell carcinoma, Multiparametric MRI, Pathology, Logistic model, Prognosis

## Abstract

**Background:**

The detection of renal cell carcinoma (RCC) has been rising due to the enhanced utilization of cross-sectional imaging and incidentally discovered lesions with adverse pathology demonstrate potential for metastasis. The purpose of our study was to determine the clinical and multiparametric dynamic contrast-enhanced magnetic resonance imaging (CEMRI) associated independent predictors of adverse pathology for cT1/2 RCC and develop the predictive model.

**Methods:**

We recruited 105 cT1/2 RCC patients between 2018 and 2022, all of whom underwent preoperative CEMRI and had complete clinicopathological data. Adverse pathology was defined as RCC patients with nuclear grade III-IV; pT3a upstage; type II papillary RCC, collecting duct or renal medullary carcinoma, unclassified RCC; sarcomatoid/rhabdoid features. The qualitative and quantitative CEMRI parameters were independently reviewed by two radiologists. Univariate and multivariate binary logistic regression analyses were utilized to determine the independent predictors of adverse pathology for cT1/2 RCC and construct the predictive model. The receiver operating characteristic (ROC) curve, confusion matrix, calibration plot, and decision curve analysis (DCA) were conducted to compare the diagnostic performance of different predictive models. The individual risk scores and linear predicted probabilities were calculated for risk stratification, and the Kaplan–Meier curve and log-rank tests were used for survival analysis.

**Results:**

Overall, 45 patients were pathologically confirmed as RCC with adverse pathology. Clinical characteristics, including gender, and CEMRI parameters, including RENAL score, tumor margin irregularity, necrosis, and tumor apparent diffusion coefficient (ADC) value were identified as independent predictors of adverse pathology for cT1/2 RCC. The clinical-CEMRI predictive model yielded an area under the curve (AUC) of the ROC curve of 0.907, which outperformed the clinical model or CEMRI signature model alone. Good calibration, better clinical usefulness, excellent risk stratification ability of adverse pathology and prognosis were also achieved for the clinical-CEMRI predictive model.

**Conclusions:**

The proposed clinical-CEMRI predictive model offers the potential for preoperative prediction of adverse pathology for cT1/2 RCC. With the ability to forecast adverse pathology, the predictive model could significantly benefit patients and clinicians alike by providing enhanced guidance for treatment planning and decision-making.

**Supplementary Information:**

The online version contains supplementary material available at 10.1186/s12957-024-03431-4.

## Introduction

Renal cell carcinoma (RCC) is a genitourinary malignancy originating from the epithelial cells of renal tubules [1]. It is the most frequent form of kidney cancer, accounting for approximately 90% of all cases and mainly including clear cell RCC (ccRCC; 70%), papillary RCC (pRCC; 10%-15%), and chromophobe RCC (chRCC; 5%) [2]. The age-standardized incidence rates of RCC have increased from 4.72/100,000 to 4.94/100,000 between 1990 and 2017, primarily due to the widespread use of advanced imaging modalities that enable the detection of small renal masses [3, 4]. The management of localized RCC has evolved significantly in recent years and was encapsulated within contemporary clinical guidelines, which recommend a range of treatment options based on tumor characteristics, patient comorbidities, and preferences [5]. The recommended treatments include surgery, active surveillance, and ablative therapies, with each tailored to specific clinical scenarios and patient factors [6].

Localized RCC pertains to T1 and T2 tumors that are confined within the kidney and can be treated with the potential for a cure. It is noteworthy that the choice of treatment is guided by the pathological characteristics of the localized RCC, such as size, tumor-node-metastasis (TNM) stage, histology subtype, tumor nuclear grade, sarcomatoid component, among others [7, 8]. For patients with low-grade, small renal masses, and indolent histology subtypes, active surveillance is a viable option due to the slow-growing nature of these tumors and the desire to avoid overtreatment [9]. However, for RCC with adverse pathological features, immediate intervention including partial nephrectomy (PN) and radical nephrectomy (RN) is typically recommended to reduce the risk of progression and metastasis [10]. Ablative therapies, such as cryoablation and radiofrequency ablation, offer alternative treatment options for patients with small, localized RCC. These minimally invasive techniques aim to destroy the tumor while preserving surrounding renal tissue, providing a balance between treating the RCC and preserving function in carefully selected patients [11].

Dynamic contrast-enhanced magnetic resonance imaging (CEMRI) is an essential imaging technique used for the assessment and treatment of localized RCC. Its utility spans across differentiating RCC subtypes, assessing tumor aggressiveness, and providing insights into patient prognosis. Compared to other imaging methods, MRI offers undeniable advantages due to its high spatial resolution, exceptional soft-tissue characterization, and non-ionizing radiation [12]. With its impressive ability to detect intracystic architecture, intracellular fat, and hemorrhage, it has become an ideal option for renal imaging in cases where other methods have proven inconclusive and for patients who cannot undergo iodinated contrast medium [12, 13]. More importantly, by combining various MRI sequences into a multiparametric study, medical professionals can more reliably investigate the potential histology of RCC [14]. The multiparametric dynamic CEMRI could provide anatomic information from T1-weighted imaging (T1WI) and T2-weighted imaging (T2WI), display macro and microscopic fat from chemical-shift imaging, evaluate tumor cell density from diffusion-weighted imaging (DWI), and exhibit tumor vascularity from CEMRI [13]. Modern multiparametric dynamic CEMRI is capable of providing a comprehensive range of qualitative, semiquantitative, and quantitative imaging information that can be directly correlated with histological subtype, tumor grade, and clinical behavior, however, there is a lack of related research in this regard. Therefore, our study aims to ascertain CEMRI parameters alongside clinical features that can be used to detect adverse pathology in cT1/2 RCC patients and develop a predictive model that can assist in determining appropriate treatment options and prognosis.

## Materials and methods

### Patient cohort

**Fig. 1:**
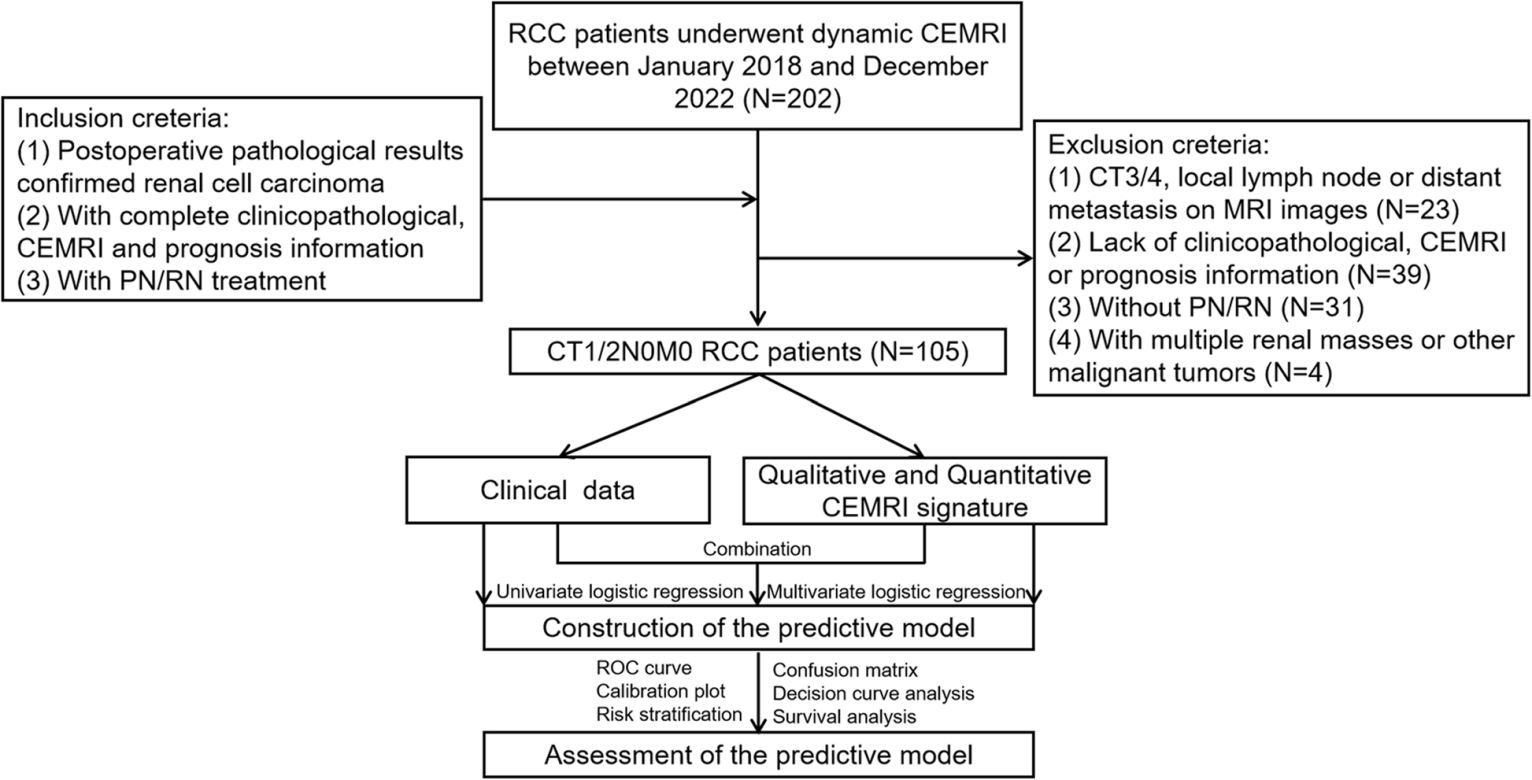
The flowchart of patient inclusion and the workflow of our study. RCC: renal cell carcinoma; CEMRI: contrast-enhanced magnetic resonance imaging; PN: partial nephrectomy; RN: radical nephrectomy; ROC: receiver operating characteristic

We retrospectively analyzed the data of the Second Hospital of Tianjin Medical University, for cT1/2 RCC patients underwent nephrectomy between January 2018 and December 2022 (*n* = 105). To be included in our analysis, patients had to meet specific criteria: (1) Patients with postoperative pathological results confirmed RCC; (2) with complete clinicopathological, CEMRI, and prognosis information; (3) with PN/RN treatment. Main exclusion criteria were as follows: (1) Patients with cT3/4, local lymph node, or distant metastasis on MRI images; (2) Lack of clinicopathological, CEMRI, or prognosis information; (3) Without PN/RN; (4) With multiple renal masses or other malignant tumors. The overall flowchart and detailed enrollment process are shown in Fig. 1.

### Clinicopathological assessment

Our study considered a total of 9 baseline information factors, [age, gender, tumor laterality, ECOG performance status, symptomatic presentation, hypertension, diabetes, smoking history, body mass index (BMI)], 2 laboratory tests [hemoglobin, neutrophil-to-lymphocyte ratio, (NLR)], 2 surgical findings (surgical approach, type of nephrectomy), and 7 pathological results (clinical tumor size, cT stage, histology subtype, surgical margin status, tumor grade, pT stage, sarcomatoid/rhabdoid component). Pathological diagnoses were determined by two experienced genitourinary pathologists after the initial surgery and the pathological results were assessed according to the 2022 WHO/ISUP classification. The RCC staging was determined by the 8^th^ edition of the American Joint Committee on Cancer (AJCC) TNM staging system. If any of the following conditions are met, the diagnosis of adverse pathology is confirmed: (1) nuclear grade III-IV; (2) upstage to pT3a; (3) non-clear cell subtypes with adverse prognosis (type II pRCC, collecting duct or renal medullary carcinoma, unclassified RCC); (4) with sarcomatoid/rhabdoid features [15].

### CEMRI examination

MRI examinations were conducted on a 3.0T Siemens MAGNETOM vida MRI scanner. Patients were scanned in the supine position using an abdominal phased-array surface coil. The following sequences were acquired before administering the contrast agent: axial and coronal T2-weighted periodically rotated overlapping parallel lines with enhanced reconstruction (PROPELLER) sequence, axial fat-suppressed T2-weighted PROPELLER sequence, and axial dual echo T1-weighted in-phase and opposed-phase sequence. Axial DWI was performed using a single-shot echo planar imaging (EPI) sequence with b values of 0, 50, 1200 s/mm^2^ (repetition time: 6000 ms, echo time: 50 ms, layer thickness: 5 mm, layer gap: 1 mm, field of view: 380 mmx284 mm). Apparent diffusion coefficient (ADC) maps were automatically generated, and the acquisition time was 5 min 30s. Contrast-enhanced MRI scans were performed on all patients. Routine MRI scans were conducted using axial fat-saturated T1-weighted volumetric interpolated breath-hold (VIBE) in-phase and opposed-phase sequences. The consecutive multi-temporal dynamic enhanced MRI scan was performed after administering a bolus of 0.1 mmol/kg of Gadobutrol at a rate of 2.0 ml/s followed by a 30 ml saline flush. The corticomedullary phase (CMP), nephrographic phase (NP), and excretory phase (EP) MRI scans occurred approximately 30-40s, 90-120s, and 5–10 min after the contrast injection, respectively.

### Qualitative and quantitative CEMRI evaluation

**Fig. 2:**
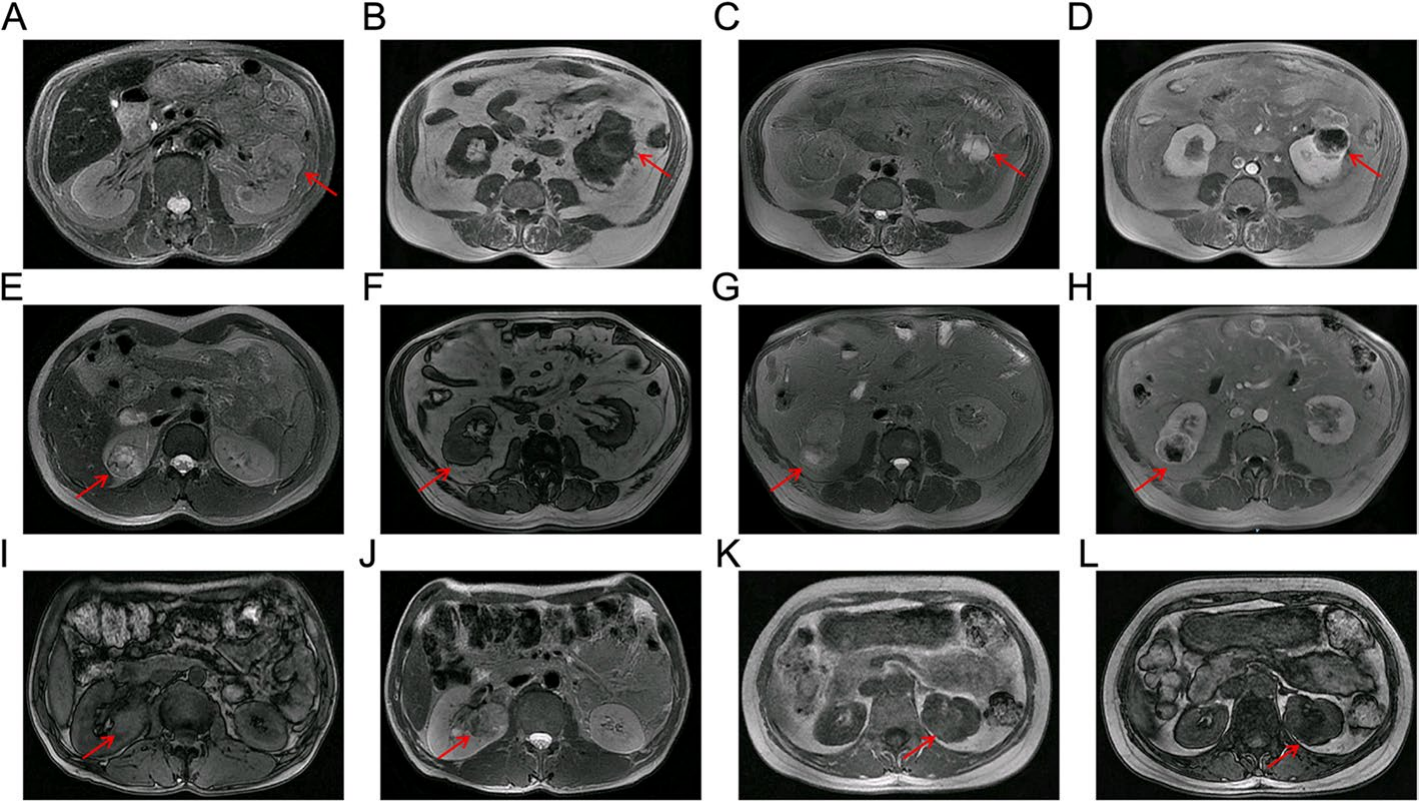
Representative qualitative CEMRI images of cT1/2 RCC. **A** A left renal tumor with an irregular margin on axial T2WI and more than 10% of the circumference of the RCC has an indistinct or blurred border between the RCC and the surrounding kidney tissue; **B**-**D** A left renal tumor with a regular well-defined cystic component on axial T1WI-PCP (low signal intensity), T2WI (high signal intensity) and T1WI-NP (no contrast enhancement); **E** A right renal tumor with pseudocapsule on axial T2WI and a rim of perilesional low signal intensity region; **F**–**H** A right renal tumor with necrosis on axial T1WI-PCP (low signal intensity), T2WI (high signal intensity) and T1WI-NP (no contrast accumulation), and the non-enhanced ill-defined component of variable MRI signal intensity; **I**-**J** A right renal tumor with hemorrhage on axial T1WI (high signal intensity) and T2WI (low signal intensity). **K**-**L** A left renal tumor with microscopic fat on axial T1-weighted in-phase and opposed-phase images. Microscopic fat is present when there is a non-linear, non-curvilinear loss of signal intensity in any part of a renal tumor on T1-weighted opposed-phase images compared to T1-weighted in-phase images. RCC: renal cell carcinoma; T1WI: T1-weighted imaging; T2WI: T2-weighted imaging; PCP: precontrast phase; CMP: corticomedullary phase; NP: nephrographic phase; EP: excretory phase

Two radiologists with 4 years and 6 years of experience in urinary system MRI imaging, independently reviewed each study on a PACS workstation and blinded to the final pathological results. In cases where the two reviewers had divergent interpretations of the qualitative CEMRI feature, a third radiologist with 16 years of experience in urological malignancies imaging was invited to examine the study and reach a consensus. To assess the qualitative CEMRI, all sessions of all sequences were meticulously reviewed, and the following features were assessed: maximal tumor diameter, exophytic/endophytic rate, distance to the collecting system, polar location, RENAL score, the regularity of tumor margin, necrosis, pseudocapsule, cystic degeneration, hemorrhage, T1 signal intensity, T2 signal intensity, microscopic fat. Typical images and detailed explanations of the above features are illustrated in Fig. 2.

**Fig. 3:**
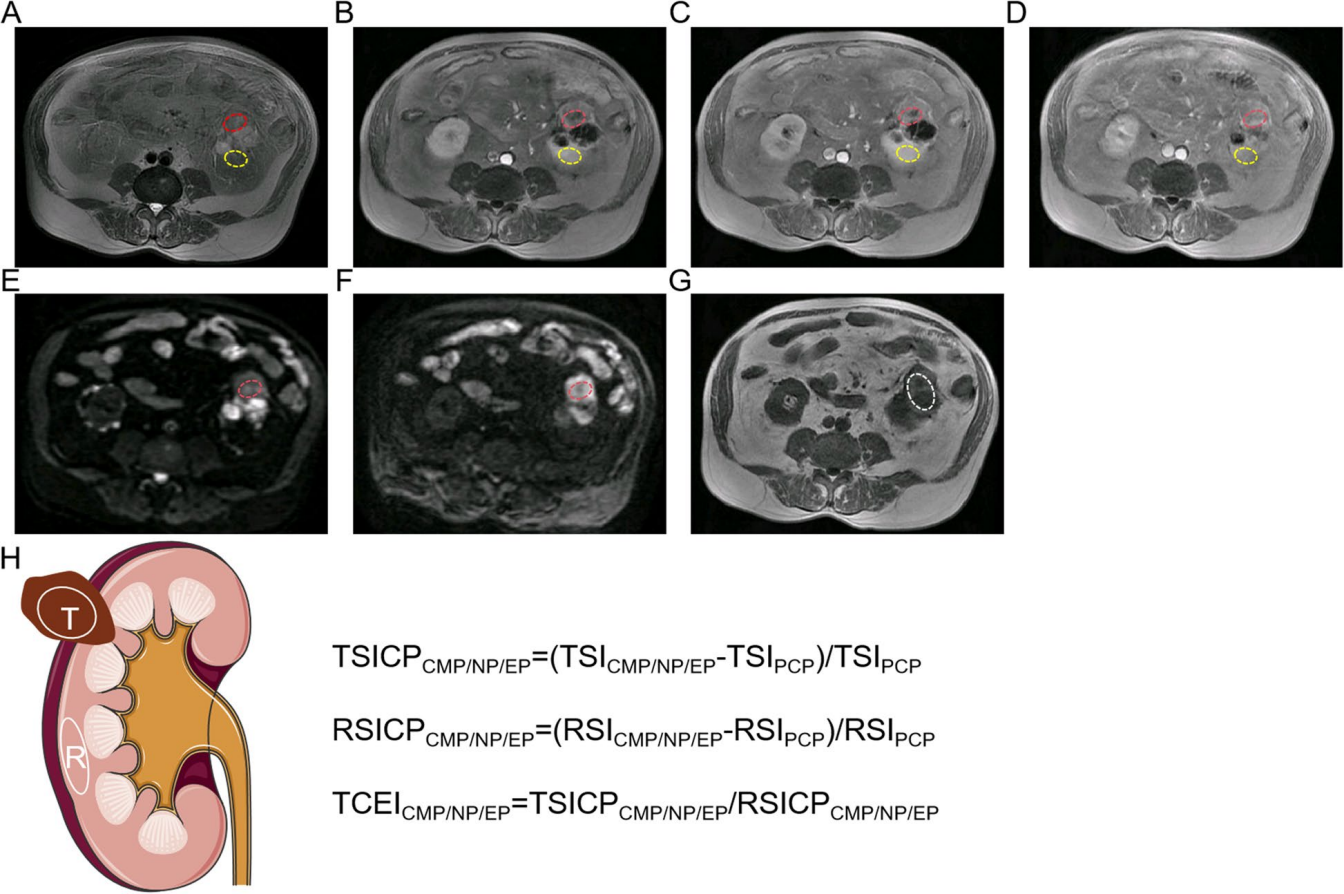
The placement method of selecting ROIs on (**A**) T1WI-PCP, (**B**) T1WI-CMP, (**C**) T1WI-NP, (**D**) T1WI-EP, (**E**) DWI (b = 0), (**F**) DWI (b = 1200), (**G**) T2WI. Red ROI: Measuring the signal intensity of the renal tumor. White ROI: Measuring the signal intensity of the renal cortex. Yellow ROI: Measuring the HDT value of the renal tumor on T2WI. **H** The schematic image and formulae for calculating TSICP, RSICP, and TCEI values of renal masses. ROI: region of interest; T1WI: T1-weighted imaging; T2WI: T2-weighted imaging; PCP: precontrast phase; CMP: corticomedullary phase; NP: nephrographic phase; EP: excretory phase; TSICP: signal intensity change percentage of renal tumor, RSICP: signal intensity change percentage of renal cortex, TCEI: tumor-to-cortex enhancement index; HDT: heterogeneous degree of tumor

Our study also recorded the signal intensity of the renal tumor and cortex region of interest (ROI) in T1WI-precontrast phase (PCP), T1WI-CMP, T1WI-NP, T1WI-EP, the signal intensity of renal tumor ROIs in DWI, and the heterogeneous degree of renal tumor (HDT) of ROIs in T2WI. The criteria for the selection of the renal tumor and cortex ROIs are as follows (Fig. 3A-F): (1) In the T1WI-CMP sequence, a single ROI was placed on the maximum and homogeneous solid-enhanced part of the renal tumor without cystic component, necrosis, hemorrhage, and perirenal adipose tissue. The same ROI selection criteria were also utilized to measure the signal intensity of the ipsilateral renal cortex, with an area of about 1cm^2^; (2) The renal tumor and cortex ROIs of other images should be consistent in the size and location with that of T1WI-PCP; (3) If there are multiple solid-enhanced regions in a renal tumor, they should be measured separately twice, and the average should be taken eventually. Contralateral renal cortex ROIs were measured in three patients, as the ipsilateral kidney was largely replaced by tumor tissue and it was unable to identify an area of about 1 cm^2^. Additionally, HDT was defined according to the standard deviation (SD) of T2WI signal intensity, and the ROIs should encompass the entire renal tumor region as much as possible, with margins 2–3 mm medial to the tumor boundary (Fig. 3G).

The present study reports the determination of the signal intensity change percentage of renal tumor (TSICP) and cortex (RSICP) and tumor-to-cortex enhancement index (TCEI) on CEMRI images, as shown in Fig. 3H. These parameters have previously been reported in a separate study [16, 17]. The formulas are as follows: ${\text{TSICP}}_{\text{CMP}/\text{NP}/\text{EP}}=\left[\left({\text{TSI}}_{\text{CMP}/\text{NP}/\text{EP}}-{\text{TSI}}_{\text{PCP}}\right)/{\text{TSI}}_{\text{PCP}}\right]$; ${\text{RSICP}}_{\text{CMP}/\text{NP}/\text{EP}}=\left[\left({\text{RSI}}_{\text{CMP}/\text{NP}/\text{EP}}-{\text{RSI}}_{\text{PCP}}\right)/{\text{RSI}}_{\text{PCP}}\right]$; ${\text{TCEI}}_{\text{CMP}/\text{NP}/\text{EP}}={\text{TSICP}}_{\text{CMP}/\text{NP}/\text{EP}}/{\text{RSICP}}_{\text{CMP}/\text{NP}/\text{EP}}$.

In these formulas, TSI_PCP/CMP/NP/EP_ and RSI_PCP/CMP/NP/EP_ represent the signal intensity of the renal tumor and cortex ROIs in the T1WI-PCP/CMP/NP/EP images, while the final TSI, RSI, DWI and HDT are the averages measured by two radiologists.

### Statistical analysis

The statistical analysis applied in this study involved describing categorical and continuous variables using frequency (percentage) and median [interquartile range (IQR)], respectively. In the analysis of categorical variables, the Chi-square test and Fisher exact test were utilized to explore the relationship between qualitative variables and the adverse pathology of RCC. For continuous variables, the Student t-test and Mann–Whitney U test were employed to assess differences between quantitative values and the adverse pathology of RCC. As there were two radiologists for quantitative CEMRI and reviewer 1 evaluated twice, the intra-observer and inter-observer interclass correlation coefficient scores (ICC, > 0.8 being regarded as good reproducibility) were performed to evaluate the agreement of quantitative CEMRI parameters.

To identify the independent predictors of adverse pathology based on a combination of clinicopathological and CEMRI characteristics, univariate and multivariate binary logistic regression analyses were conducted. Variation Inflation Factors (VIF, > 5 being regarded as significant) were conducted to assess the collinearity for significant variables after the univariate logistic regression. The performance of different predictive models including accuracy, reliability, calibration, and clinical net benefit was compared using the receiver operating characteristic (ROC) curve, confusion matrix, calibration plots, and decision curve analysis (DCA), respectively. The linear predicted probabilities of individual adverse pathology in different predictive models were also calculated according to the significant predictors and the regression coefficients, and the patient population was stratified into low-risk, medium-risk, and high-risk groups. Furthermore, we also calculated the actual probability of adverse pathology in different groups and performed the survival analysis of recurrence free survival (RFS). Statistical significance was set at *P* < 0.05, and the analysis was conducted using SPSS 22.0 and R software (version 4.3.1).

## Results

### Patient features

Table 1 provides an overview of the clinicopathological characteristics of cT1/2 RCC patients. Of the 105 patients with pathologically confirmed cT1/2 RCC, 45 individuals showed adverse pathology, accounting for 42.9%. Among the 45 cT1/2 RCC patients with adverse pathology, 38 (84.4%) showed III-IV tumor nuclear grade, 18 (40.0%) had pT3a RCC, 3 (6.7%) suffered from type II pRCC, collecting duct, renal medullary carcinoma, unclassified RCC, and 4 (8.9%) had RCC with sarcomatoid/rhabdoid component. The median age of all patients was 62.0 years with 63 (60.0%) of the patients being male, and the median tumor size of all RCCs was 4.4cm. Comparative analysis showed that gender, type of nephrectomy, clinical tumor size, and cT stage were statistically different between cT1/2 RCC patients with and without adverse pathology.

**Table 1:** Clicopathological characteristics of patients with cT1/2 RCC

Characteristics	Overall (*N* = 105)	Without adverse pathology (*N* = 60)	With adverse pathology (*N* = 45)	*P*
**Clinical findings**				
Age (years)	62.0 (53.0–67.0)	61.0 (51.0–67.8)	64.0 (55.0–66.0)	0.200
Gender: male	63.0 (60.0)	30.0 (50.0)	33.0 (73.3)	0.016
Laterality: left	46.0 (43.8)	23.0 (38.3)	23.0 (51.1)	0.192
ECOG performance status grade: 2–4	7.0 (6.7)	4.0 (6.7)	3.0 (6.7)	1.000^#^
Symptomatic presentation	45.0 (42.9)	24.0 (40.0)	21.0 (46.7)	0.495
Hypertension	43.0 (41.0)	22.0 (36.7)	21.0 (46.7)	0.302
Diabetes	15 (14.3)	6 (10.0)	9.0 (20.0)	0.147
Smoking history	27.0 (25.7)	14.0 (23.3)	13.0 (28.9)	0.519
BMI (kg/m^2^)	24.5 (22.8–27.6)	24.5 (22.6–27.4)	24.9 (23.0–28.3)	0.313
Hemoglobin (g/L)	132.0 (121.0–143.0)	133.5 (123.5–144.8)	128.0 (114.5–141.5)	0.063
NLR	1.9 (1.3–2.6)	1.8 (1.3–2.3)	2.0 (1.2–3.2)	0.168
**Surgical findings**				
Surgical approach				0.333
Open	9.0 (8.6)	5.0 (8.3)	4.0 (8.9)	
Laparoscopic	79.0 (75.2)	48.0 (80.0)	31.0 (68.9)	
Robotic	17.0 (16.2)	7.0 (11.7)	10.0 (22.2)	
Type of nephrectomy				< 0.001
Partial nephrectomy	56.0 (53.3)	41.0 (68.3)	15.0 (33.3)	
Radical nephrectomy	49.0 (46.7)	19.0 (31.7)	30.0 (66.7)	
**Pathologic findings**				
Clinical tumor size (cm)	4.4 (3.0–6.3)	3.9 (2.6–5.3)	5.5 (3.7–7.3)	0.003
CT stage				0.042^#^
T1a	48.0 (45.7)	33.0 (55.0)	15.0 (33.3)	
T1b	38.0 (36.2)	21.0 (35.0)	17.0 (37.8)	
T2a	15.0 (14.3)	5.0 (8.3)	10.0 (22.2)	
T2b	4.0 (3.8)	1.0 (1.7)	3.0 (6.7)	
Histology subtype				0.220^#^
Clear cell RCC	84.0 (80.0)	48.0 (80.0)	36.0 (80.0)	
Papillary RCC	13.0 (12.4)	7.0 (11.7)	6.0 (13.3)	
Chromphobe RCC	4.0 (3.8)	4.0 (6.7)	0.0 (0.0)	
Others	4.0 (3.8)	1.0 (1.7)	3.0 (6.7)	
Surgical margin: positive	3.0 (2.9)	2.0 (3.3)	1.0 (2.2)	0.735
Adverse pathology				
Tumor grade: III-IV	38.0 (36.2)	0.0 (0.0)	38.0 (84.4)	
PT3a upstage	18.0 (17.1)	0.0 (0.0)	18.0 (40.0)	
type II pRCC, collecting duct, renal medullary carcinoma, unclassified RCC	3.0 (2.9)	0.0 (0.0)	3.0 (6.7)	
Sarcomatoid/rhabdoid component	4.0 (3.8)	0.0 (0.0)	4.0 (8.9)	

*BMI* body mass index, *RCC* renal cell carcinoma, *pRCC* papillary renal cell carcinoma, *NLR* neutrophil-to-lymphocyte ratio

^#^*P* values were calculated by Fisher exact tests

### Analysis of CEMRI characteristics

The qualitative and quantitative CEMRI parameters of cT1/2 RCC patients are presented in Table 2. Of all the qualitative CEMRI parameters, cT1/2 RCC patients with adverse pathology had larger tumor diameter (*P* = 0.021), closer distance to the collecting system (*P* < 0.001), higher rate of RCCs with more than 50% crossing the polar line, crossing the axial renal midline or entirely between the polar lines (*P* = 0.039), higher RENAL score (*P* < 0.001), irregular tumor margin (*P* < 0.001), higher rate of tumor necrosis(*P* < 0.001) and pseudocapsule (*P* = 0.009) compared to those without adverse pathology.

**Table 2:** Qualitative and quantitative CEMRI parameters of patients with cT1/2 RCC

Characteristics	Overall(*N* = 105)	Without adverse pathology (*N* = 60)	With adverse pathology (*N* = 45)	*P*
**Qualitative MRI parameters**				
Maximal tumor diameter (cm)				0.021
≤ 4	48.0 (45.7)	33.0 (55.0)	15.0 (33.3)	
> 4- < 7	38.0 (36.2)	21.0 (35.0)	17.0 (37.8)	
≥ 7	19.0 (18.1)	6.0 (10.0)	13.0 (28.9)	
Exophytic/endophytic rate				0.864
≥ 50%	52.0 (49.5)	30.0 (50.0)	22.0 (48.9)	
< 50%	42.0 (40.0)	23.0 (38.3)	19.0 (42.2)	
Endophytic	11.0 (10.5)	7.0 (11.7)	4.0 (8.9)	
Distance to the collecting system (mm)				< 0.001^#^
> 7	55.0 (52.4)	41.0 (68.3)	14.0 (31.1)	
4–7	8.0 (7.6)	2.0 (3.3)	6.0 (13.3)	
≤ 4	42.0 (40.0)	17.0 (28.3)	25.0 (55.6)	
Polar location				0.039
Entirely above or below the polar line	24.0 (22.9)	19.0 (31.7)	5.0 (11.1)	
Cross the polar line	39.0 (37.1)	21.0 (35.0)	18.0 (40.0)	
> 50% crosses the polar line, crosses the axial renal midline or entirely between the polar lines	42.0 (40.0)	20.0 (33.3)	22.0 (48.9)	
RENAL score				< 0.001
Low (4–6)	37.0 (35.2)	29.0 (48.3)	8.0 (17.8)	
Intermediate (7–9)	50.0 (47.6)	27.0 (45.0)	23.0 (51.1)	
High (10–12)	18.0 (17.1)	4.0 (6.7)	14.0 (31.1)	
Tumor margin irregularity	39.0 (37.1)	9.0 (15.0)	30.0 (66.7)	< 0.001
Necrosis	29.0 (27.6)	7.0 (11.7)	22.0 (48.9)	< 0.001
Pseudocapsule	48.0 (45.7)	34.0 (56.7)	14.0 (31.1)	0.009
Cystic degeneration				0.644^#^
≤ 25%	89.0 (84.8)	50.0 (83.3)	39.0 (86.7)	
25%-75%	11.0 (10.5)	6.0 (10.0)	5.0 (11.1)	
> 75%	5.0 (4.8)	4.0 (6.7)	1.0 (2.2)	
Haemorrhage	69.0 (65.7)	40.0 (66.7)	29.0 (64.4)	0.838
T1 signal intensity				0.075
Hypointense	23.0 (21.9)	14.0 (23.3)	9.0 (20.0)	
Isointense	66.0 (62.9)	33.0 (55.0)	33.0 (73.3)	
Hyperintense	16.0 (15.2)	13.0 (21.7)	3.0 (6.7)	
T2 signal intensity				0.187
Hypointense	5.0 (4.8)	2.0 (3.3)	3.0 (6.7)	
Isointense	7.0 (6.7)	2.0 (3.3)	5.0 (11.1)	
Hyperintense	93.0 (88.6)	56.0 (93.3)	37.0 (82.2)	
Microscopic fat	41.0 (39.0)	27.0 (45.0)	14.0 (31.1)	0.163
**Quantitative MRI parameters**				
Tumor ADC value (mm^2^/s)	1556.7 (1180.2–1867.4)	1616.9 (1383.9–1967.3)	1433.6 (1056.7–1629.4)	0.007
T2-HDT	213.0 (162.0–276.0)	219.0 (168.3–287.3)	178.0 (149.5–265.0)	0.073
Corticomedullary phase				
TSICP-CMP	1.3 (0.9–2.0)	1.5 (0.9–2.1)	1.1 (0.8–1.9)	0.129
TCEI-CMP	0.8 (0.5–1.2)	1.0 (0.6–1.3)	0.7 (0.4–1.0)	0.004
Nephrographic phase				
TSICP-NP	1.4 (0.9–2.1)	1.5 (1.0–2.2)	1.1 (0.9–1.9)	0.128
TCEI-NP	0.8 (0.6–1.2)	1.0 (0.6–1.3)	0.7 (0.5–1.0)	0.007
Excretory phase				
TSICP-EP	1.2 (0.9–2.0)	1.5 (1.0–2.2)	1.0 (0.8–1.8)	0.125
TCEI-EP	0.8 (0.5–1.2)	0.9 (0.6–1.3)	0.7 (0.5–1.0)	0.009

*RCC* renal cell carcinoma, *ADC* apparent diffusion coefficient, *HDT* heterogeneous degree of tumor, *TSICP* signal intensity change percentage of renal tumor, *TCEI* tumor-to-cortex enhancement index, *CMP* corticomedullary phase, *NP* nephrographic phase, *EP* excretory phase

As the TSI, RSI, DWI, and HDT were measured by reviewer 1 twice and also by two reviewers, the intra-observer and inter-observer ICC scores were shown in Supplementary Table 1, demonstrating perfect reliability. The intra-observer ICC scores for radiologist 1 range from 0.899 to 0.998, and the inter-observer ICC scores between two radiologists range from 0.893 to 0.998. Among all quantitative MRI parameters, RCC patients with adverse pathology had lower tumor ADC values (*P* = 0.007), and lower TCEI in CMP (*P* = 0.004), NP (0.007), and EP (*P* = 0.009) with statistical significance than those without adverse pathology (Table 2).

### Construction of predictive models

The clinicopathological and CEMRI features independently correlated with adverse pathology of RCC were identified by using logistic regression analyses, respectively. As illustrated in Supplementary Table 2, gender: male (OR = 2.730, *p* = 0.023) and clinical tumor size (OR = 1.300, *p* = 0.006) were independent risk factors of adverse pathology for cT1/2 RCC among all clinicopathological features. In addition, five qualitative and four quantitative CEMRI features were found to be significantly different between two groups in the univariate logistic regression analysis, including distance to the collecting system (*P* = 0.001), RENAL score (*P* = 0.001), tumor margin irregularity (*P* < 0.001), necrosis (*P* < 0.001), pseudocapsule (*P* = 0.010), tumor ADC value (*P* = 0.010), TCEI-CMP (*P* = 0.014), TCEI-NP (*P* = 0.019) and TCEI-EP (*P* = 0.024) (Supplementary Table 3). Subsequently, the collinearity test was performed for the nine variables selected from the univariate analysis, and TCEI-NP and TCEI-EP were excluded from the following analysis due to collinearity. The multivariate logistic regression analysis indicated that RENAL score (*P* = 0.021), tumor margin irregularity (OR = 7.109, *P* < 0.001), necrosis (OR = 5.549, *p* = 0.005) and tumor ADC value (OR = 0.998, *p* = 0.006) were independent predictors of adverse pathology for cT1/2 RCC. Finally, the multivariate logistic regression analyses were performed for 2 independent clinical features and 4 independent CEMRI signatures, and gender: male (OR = 6.727, *p* = 0.005), RENAL score (*P* = 0.010), tumor margin irregularity (OR = 9.607, *P* < 0.001), necrosis (OR = 9.115, *p* = 0.003) and tumor ADC value (OR = 0.999, *p* = 0.020) were independent predictors of adverse pathology in the clinical-CEMRI perdictive model (Table 3).

**Table 3:** Multivariate logistic regression analyses of clinical-CEMRI parameters for adverse pathology in cT1/2 RCC patients

Variables	Multivariate			
	OR	95%CI		*P* value
**Clinical findings**				
Gender: male	**6.727**	**1.765–25.630**		**0.005**
Clinical tumor size (cm)	0.808	0.577–1.132		0.215
**Qualitative MRI parameters**				
RENAL score				**0.010**
Low (4–6)	**1 (Reference)**			
Intermediate (7–9)	**3.160**	**0.897–11.140**		**0.073**
High (10–12)	**16.477**	**2.639–102.858**		**0.003**
Tumor margin irregularity	**9.607**	**2.742–33.662**		** < 0.001**
Necrosis	**9.115**	**2.091–39.735**		**0.003**
**Quantitative MRI parameters**				
Tumor ADC value (mm2/s)	**0.999**	**0.998–1.000**		**0.020**

*RCC* renal cell carcinoma, *ADC* apparent diffusion coefficient

### Performance of different models

**Fig. 4:**
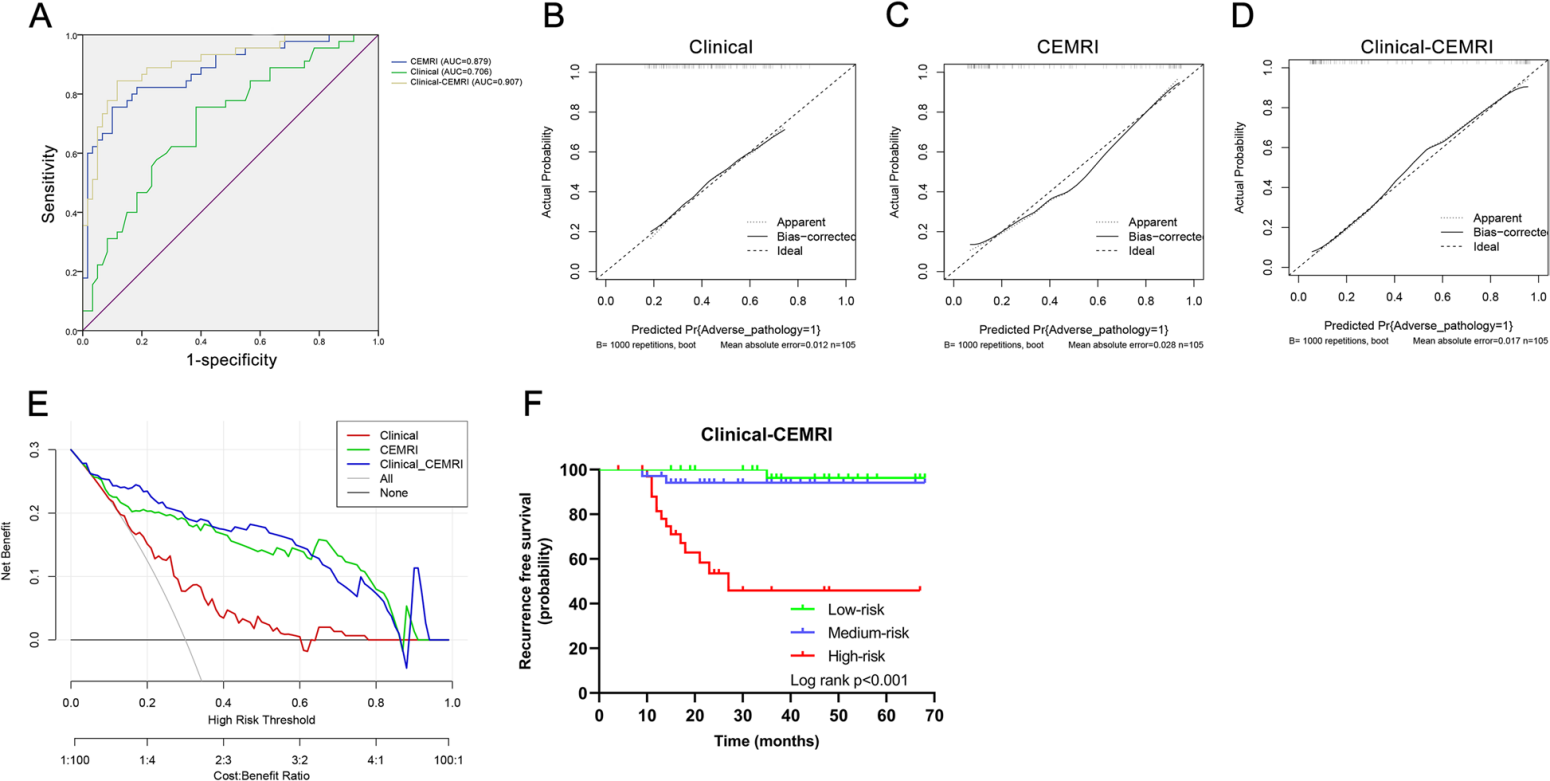
**A** ROC curve, **B**-**D** calibration plots, and (**E**) DCA of the clinical characteristics, CEMRI parameters, and clinical-CEMRI combination models for predicting adverse pathology of cT1/2 RCC. **F** Kaplan–Meier curve of RFS after risk stratification using individual linear predictive probability of adverse pathology

The discrimination of different predictive models was firstly evaluated by ROC curves, and the area under the curve (AUC) of the ROC curve was 0.706 for the clinical predictive model, 0.879 for the CEMRI signature model, and 0.907 for the clinical-CEMRI combination model, respectively (Fig. 4A, Supplementary Table 4). Moreover, the accuracy, sensitivity, specificity, PPV, NPV, and F1-score for the clinical-CEMRI combination predictive model were 0.857, 0.778, 0.917, 0.875, 0.846, and 0.824, respectively, which showed the best diagnostic performance of the adverse pathology for cT1/2 RCC compared to other models (Supplementary Table 4). The Hosmer–Lemeshow tests showed P values of 0.826, 0.614, and 0.480 for clinical, CEMRI, and clinical-CEMRI predictive models, respectively, and the calibration plots exhibited excellent concordance between the predicted and actual probability of adverse pathology in these predictive models (Fig. 4B-D). The DCA of clinical, CEMRI, and clinical-CEMRI predictive models was presented in Fig. 4E, and the clinical-CEMRI model obtained the best clinical net benefit.

### Risk stratification and survival analysis of the final predictive model

The risk scores and linear predictive probabilities were calculated for each patient by obtained characteristics and weights corresponding to their regression coefficients. The formulas are as follows:

$$\mathrm{Risk}\;\mathrm{score}=\sum_{\mathrm i=1}^{\mathrm n}\;\mathrm{coefficients}\left[\mathrm i\right]\times\mathrm{Feature}\left[\mathrm i\right]+\mathrm{Intercept}$$

$$\mathrm{Individual}\;\mathrm{predicted}\;\mathrm{probability}=1/\left(1+\mathrm e^{-\mathrm{risk}\;\mathrm{score}}\right)$$

$$\mathrm{Risk}\;\mathrm{score}\left(\mathrm{Clinical}\right)=1.004\times1\left(\mathrm{male}\right)/0\;\left(\mathrm{female}\right)+0.263\times\mathrm{tumor}\;\mathrm{size}-2.184$$

$$\begin{array}{l}\mathrm{Risk}\;\mathrm{score}\left(\mathrm{CEMRI}\right)=1.714\times1\left(\mathrm{with}\;\mathrm{necrosis}\right)/0\left(\mathrm{without}\;\mathrm{necrosis}\right)\\ +1.961\times1\left(\mathrm{irregular}\;\mathrm{tumor}\;\mathrm{margin}\right)/0\left(\mathrm{regular}\;\mathrm{tumor}\;\mathrm{margin}\right)\\ +0\left(\mathrm{RENAL}\;\mathrm{score}:4-6\right)/0.811\left(\mathrm{RENAL}\;\mathrm{score}:7-9\right)/2.441\left(\mathrm{RENAL}\;\mathrm{score}:10-12\right)\\ -0.002\times\mathrm{tumor}\;\mathrm{ADC}\;\mathrm{value}+0.030\end{array}$$

$$\begin{aligned} \mathrm{Risk}\;\mathrm{score}\;\left(\mathrm{Clinical}-\mathrm{CEMRI}\right)=1.906\times1\left(\mathrm{male}\right)/0\left(\mathrm{female}\right)+2.210\times1\left(\mathrm{with}\;\mathrm{necrosis}\right)\\ /0\;\left(\mathrm{without}\;\mathrm{necrosis}\right)+2.262\times1\left(\mathrm{irregular}\;\mathrm{tumor}\;\mathrm{margin}\right)/0\left(\mathrm{regular}\;\mathrm{tumor}\;\mathrm{margin}\right)\\ +0\left(\mathrm{RENAL}\;\mathrm{score}:4-6\right)/1.151\left(\mathrm{RENAL}\;\mathrm{score}:7-9\right)/2.802\left(\mathrm{RENAL}\;\mathrm{score}:10-12\right)\\ -0.001\times\mathrm{tumor}\;\mathrm{ADC}\;\mathrm{value}-1.832\end{aligned}$$

Then, we divided all patients into low-risk, medium-risk, and high-risk groups according to the linear predictive probabilities calculated from the predictive models, respectively. The probabilities of adverse pathology in the low-risk, medium-risk and high-risk groups were 25.7% (9/35), 40.0% (14/35), and 62.9% (22/35) in the clinical predictive model; 8.6% (3/35), 34.3% (12/35), and 85.7% (30/35) in the CEMRI predictive model; 8.6% (3/35), 31.4% (11/35), and 88.6% (31/35) in the clinical-CEMRI predictive model. The clinical-CEMRI predictive model also demonstrated the best risk stratification ability. Since adverse pathology is an important risk factor for RCC patients, the study also investigated the RFS of all enrolled patients. The probabilities of 1-year and 3-year RFS predicted via the clinical-CEMRI model were 100%, 97.1%, 81.4%, and 96.3%, 94.1%, 45.9% in the low-risk, medium-risk, and high-risk groups, respectively. The Kaplan–Meier curve and log-rank tests are shown in Fig. 4F, and the RFS of patients in high-risk groups was worse than other groups with statistical significance (*P* < 0.001).

## Discussion

Percutaneous biopsy of renal masses is a minimally invasive technique that may provide an option for the pathological diagnosis of renal tumors [18]. However, the possibility of inaccurate tumor pathological diagnosis due to inappropriate sampling, and procedural complications such as bleeding, infection, and tumor seeding along the needle track may further limit the use of it, particularly in high-risk patients or those with comorbidities [19]. Consequently, the utilization of non-invasive imaging techniques for the histopathological analysis of localized RCC is considered to provide more benefits compared to invasive methods [20]. The expanding range of treatment choices for localized RCC has resulted in a wider application of imaging techniques, extending from the identification of tumors to the prediction of tumor behavior [21]. MRI is highly appropriate for the prediction of pathological diagnosis due to its detailed insights of superior soft-tissue differentiation and innovative methods like DWI and dynamic CEMRI [21, 22]. Identifying MRI parameters correlated to adverse RCC characteristics could aid in correctly choosing localized RCC patients suitable for conservative treatment. Therefore, in this study, we developed a predictive model that could accurately predict adverse pathology for cT1/2 RCC at final pathology. Our model was constructed based on multiparametric dynamic CEMRI parameters and clinical predictors with excellent discrimination, accuracy, and value in clinical settings.

Previous studies have reported the relationship between MRI signature and single pathological finding of RCC, including histology subtype, tumor nuclear grade, pathological stage, sarcomatoid differentiation, etc. Serter et al. demonstrated that mean tumor ADC value, contrast enhancement rate, and contrast enhancement index values of ccRCC were significantly higher than those of non-ccRCC [23]. Aydogan et al. also found that SICP-EP, T2-weighted HASTE scale score, TCEI-CMP and TCEI-NP, TCEI-EP, tumor ADC value were the highest sensitivity and specifcity rates in the differentiation of ccRCC from non-ccRCC, respectively [17]. In addition, multiparametric MRI has proven to be accurate in differentiating low-grade ccRCC from high-grade ccRCC, and larger tumor size, lower parenchymal wash-in indices, and lower ADC ratios were observed in high-grade ccRCC significantly [24]. Similarly, features like larger size, retroperitoneal vascular collaterals, intratumoral necrosis, and renal vein thrombosis were also proven to be associated with high-grade ccRCC [25]. The excellent predictive value of MRI for T3a pathological features, including sinus fat invasion, perirenal fat invasion, and venous involvement has been reported in several studies [26, 27]. However, the research about MRI related predictors for cT1/2 RCC upstaging to pT3a is rare, with most studies based on computed tomography (CT) images, and these studies have found that tumor size, necrosis, and irregular tumor margins are closely associated with pT3a upstage [28, 29]. Additionally, the usefulness of MRI in distinguishing RCC with sarcomatoid differentiation was also investigated, emphasizing conspicuously low SI areas on T2WI as indicative of sarcomatoid RCC [30]. Since the aggressive pathological features mentioned above have a negative impact on the prognosis of localized RCC patients, our study is the first to combine the above pathological features as the outcome variable and combine the MRI parameters with clinical features as the independent variables. We found that tumor margin irregularity, higher RENAL score, necrosis, lower tumor ADC value, and male gender were independent predictors of adverse pathology for cT1/2 RCC, which is in agreement with previous studies generally [24, 25, 30–33].

Derived from DWI, ADC values of RCC furnish crucial insights into tumor density and the microenvironment. The lower tumor ADC values signify more restricted diffusion of water molecules within the tumor tissue, which can be attributed to high cellular density, cellular structure changes, and microenvironment changes [34]. The relationship between lower tumor ADC value and adverse pathological features, including malignancy, higher tumor nuclear grade, and aggressive histology subtype has been proven by previous studies, and we also identified it as the independent predictor of adverse pathology for cT1/2 RCC [35, 36]. Tumor margin irregularity, necrosis, and RENAL score are common qualitative imaging signatures, which can be evaluated by different imaging modalities, and have been reported associated with adverse pathology of RCC [15, 32, 33]. With the superior soft-tissue contrast and functional imaging techniques, multiparametric dynamic CEMRI is more advantageous for evaluating necrosis, delineation of RCC margins, and anatomical structures, as it allows for detailed assessment of tumor composition, perfusion, and cellular density [12, 37]. Consistent with the existing literature, our study also demonstrated the predictive value of these qualitative MRI features for adverse pathology in cT1/2 RCC patients. The clinical characteristics, including male, have been included in the predictive model for the prediction of unfavorable pathology in cT1 RCC and played a significant role in the estimation of aggressive histology for pT1/2 RCC patients [33, 38].

To our acknowledgment, several nomogram models have been constructed to predict adverse pathology for RCC. Karlo et al. developed a nomogram that combined CT features with clinical data to predict indolent renal tumors, achieving a concordance index of 0.823 to 0.829 upon internal and external validation, and necrosis, calcification, adjacency to renal sinus fat, invasion of the renal vein and collecting system, multicystic tumor structure, and nodular enhancement were significantly correlated with aggressive RCC [39]. Furthermore, Deng et al. focused on endophytic RCC patients and developed a nomogram for the prediction of unfavorable pathology, significantly leveraging factors like BMI, NLR, and R score, with the AUC of the nomogram model of 0.808 [40]. Since NLR is one of the most commonly studied hematological ratios and is generally used to predict the inflammatory state of tumors and overall prognosis in patients, We included it as the only haematological rate indicator. In addition, Ball et al. identified gender, tumor size, and RENAL score as preoperative predictors associated with unfavorable pathology for cT1a RCC treated with PN, assisting in risk stratification and management decisions [41]. Since most of the nomograms are based on CT images, we are the first study to make full use of the multiparametric dynamic CEMRI. The AUC of our model was 0.907, which was significantly higher than other models.

However, there are still limitations in our study. First, this was a retrospective single-institution study with a small sample size, because CEMRI is mostly used in RCC patients who cannot undergo contrast-enhanced computed tomography (CECT) or as a complementary method for other imaging modalities that could not provide a definitive diagnosis. Therefore, our study may have selection bias and need further external large sample size validation. Second, in our study, the quantitative MRI parameters were evaluated based on 2D measurements and ignored the benefits of efficient enhanced feature extraction, structural, and texture analysis in 3D image analysis. In future studies, we will attempt to incorporate 3D reconstruction information and texture analysis into the study. Third, we developed ROI placement criteria based on previous literature and manually placed ROIs on the PACS workstation. To demonstrate the generalisability of our study, the raw CEMRI data should be downloaded and the feasibility of our predictive model should be validated on other available software for imaging processing.

## Conclusion

In conclusion, the predictive model integrating CEMRI signature and clinical characteristics could effectively predict adverse pathology in cT1/2 RCC patients prior to surgery. Hence, the MRI-based predictive model could serve as a dependable and non-invasive instrument for aiding clinical decisions, offering significant promise for personalized treatment methods in cT1/2 RCC.

### Supplementary Information

Supplementary Material 1.

## Data Availability

The datasets generated and/or analyzed during the current study are available from the corresponding author on reasonable request.

## Abbreviations

ADC: Apparent diffusion coefficient
AJCC: American Joint Committee on Cancer
AUC: Area under the curve
BMI: Body mass index
CCRCC: Clear cell RCC
CECT: Contrast-enhanced computed tomography
CEMRI: Contrast-enhanced magnetic resonance imaging
CHRCC: Chromophobe RCC
CMP: Corticomedullary phase
DCA: Decision curve analysis
DWI: Diffusion-weighted imaging
EP: Excretory phase
EPI: Echo planar imaging
HDT: Heterogeneous degree of tumor
ICC: Interclass correlation coefficient
IQR: Interquartile range
NLR: Neutrophil-to-lymphocyte ratio
NP: Nephrographic phase
PCP: Precontrast phase
PN: Partial nephrectomy
PRCC: Papillary RCC
PROPELLER: Periodically rotated overlapping parallel lines with enhanced reconstruction
RCC: Renal cell carcinoma
RFS: Recurrence free survival
RN: Radical nephrectomy
ROC: Receiver operating characteristic
ROI: Region of interest
RSICP: Signal intensity change percentage of renal cortex
SD: Standard deviation
T1WI: T1-weighted imaging
T2WI: T2-weighted imaging
TCEI: Tumor-to-cortex enhancement index
TNM: Tumor-node-metastasis
TSICP: Signal intensity change percentage of renal tumor
VIBE: Volumetric interpolated breath-hold
VIF: Variation Inflation factor
