# Supplementary material for: Clinical T1/2 renal cell carcinoma: multiparametric dynamic contrast-enhanced MRI features-based model for the prediction of individual adverse pathology
Source: World J Surg Oncol. 2024 Jun 1;22:145. doi: 10.1186/s12957-024-03431-4 (PMC11143715; doi:10.1186/s12957-024-03431-4)
Supplement: Supplementary file 1 — Supplementary Material 1. [file 12957_2024_3431_MOESM1_ESM.docx]

| Supplementary Table 1.Inter-observer and intra-observer reproducibility of CEMRI features | | |
| --- | --- | --- |
| Quantitative CEMRI parameters | Inter-observer ICC | Intra-observer ICC |
| T1-TSI-PCP | 0.983 | 0.986 |
| T1-TSI-CMP | 0.992 | 0.987 |
| T1-TSI-NP | 0.991 | 0.976 |
| T1-TSI-EP | 0.990 | 0.967 |
| T1-RSI-PCP | 0.988 | 0.990 |
| T1-RSI-CMP | 0.995 | 0.994 |
| T1-RSI-NP | 0.992 | 0.992 |
| T1-RSI-EP | 0.993 | 0.991 |
| T2-HDT | 0.946 | 0.990 |
| DWI-TSI (b=0) | 0.899 | 0.893 |
| DWI-TSI (b=1200) | 0.992 | 0.998 |
| DWI-RSI (b=0) | 0.998 | 0.995 |
| DWI-RSI (b=1200) | 0.951 | 0.994 |
| RCC: renal cell carcinoma, HDT: heterogeneous degree of tumor, TSI: signal intensity of renal tumor, RSI: signal intensity of renal cortex, CMP: corticomedullary phase, NP: nephrographic phase, EP: excretory phase, ICC: interclass correlation coefficient | | |

| Supplementary Table 2. Univariate and multivariate logistic regression analyses of clicopathological characteristics for adverse pathology in cT1/2 RCC patients. | | | | | | | | | |
| --- | --- | --- | --- | --- | --- | --- | --- | --- | --- |
| Variables | Univariate | | | | | Multivariate | | | |
|  | OR | | 95%CI | | P value | OR | 95%CI | | P value |
| **Clinical findings** |  |  |  |  |  |  |  |  |  |
| Age (years) | 1.022 | | 0.988-1.058 | | 0.201 |  |  |  |  |
| Gender: male | **2.750** | | **1.196-6.321** | | **0.017** | **2.730** | **1.147-6.496** | | **0.023** |
| Laterality: left | 1.682 | | 0.769-3.678 | | 0.193 |  |  |  |  |
| ECOG performance status grade: 2-4 | 1.000 | | 0.212-4.709 | | 1.000 |  |  |  |  |
| Symptomatic presentation | 1.312 | | 0.601-2.866 | | 0.495 |  |  |  |  |
| Hypertension | 1.511 | | 0.688-3.319 | | 0.303 |  |  |  |  |
| Diabetes | 2.250 | | 0.737-6.867 | | 0.154 |  |  |  |  |
| Smoking history | 1.335 | | 0.554-3.216 | | 0.520 |  |  |  |  |
| BMI (kg/m2) | 1.070 | | 0.939-1.221 | | 0.310 |  |  |  |  |
| Hemoglobin (g/L) | 0.980 | | 0.959-1.001 | | 0.067 |  |  |  |  |
| NLR | 1.124 | | 0.958-1.319 | | 0.152 |  |  |  |  |
| Clinical tumor size (cm) | **1.302** | | **1.083-1.567** | | **0.005** | **1.300** | **1.077-1.570** | | **0.006** |
| **Surgical findings** |  |  |  |  |  |  |  |  |  |
| Surgical approach |  | |  | | 0.343 |  |  |  |  |
| Open | 1 (Reference) | | | |  |  |  |  |  |
| Laparoscopic | 0.807 | | 0.201-3.242 | |  |  |  |  |  |
| Robotic | 1.786 | | 0.349-9.127 | |  |  |  |  |  |
| BMI: body mass index, RCC: renal cell carcinoma, NLR: neutrophil-to-lymphocyte ratio. | | | | | | | | | |

| Supplementary Table 3. Univariate and multivariate logistic regression analyses of qualitative and quantitative CEMRI parameters for adverse pathology in cT1/2 RCC patients. | | | | | | | | | |
| --- | --- | --- | --- | --- | --- | --- | --- | --- | --- |
| Variables | Univariate | | | | | Multivariate | | | |
|  | OR | | 95%CI | | P value | OR | 95%CI | | P value |
| **Qualitative MRI parameters** |  |  |  |  |  |  |  |  |  |
| Exophytic/endophytic rate |  |  |  |  | 0.864 |  |  |  |  |
| ≥50% | 1 (Reference) | | | |  |  |  |  |  |
| <50% | 1.126 | | 0.496-2.557 | | 0.776 |  |  |  |  |
| Endophytic | 0.779 | | 0.203-2.994 | | 0.716 |  |  |  |  |
| Distance to the collecting system (mm) |  |  |  |  | **0.001** |  |  |  |  |
| >7 | **1 (Reference)** | | | |  |  |  |  |  |
| 4-7 | **8.786** | | **1.587-48.646** | | **0.013** |  |  |  |  |
| ≤4 | **4.307** | | **1.814-10.225** | | **0.001** |  |  |  |  |
| Polar location |  |  |  |  | 0.050 |  |  |  |  |
| Entirely above or below the polar line | 1 (Reference) | | | |  |  |  |  |  |
| Cross the polar line | 3.257 | | 1.012-10.485 | | 0.048 |  |  |  |  |
| >50% crosses the polar line crosses the axial renal midline or entirely between the polar lines | 4.180 | | 1.315-13.285 | | 0.015 |  |  |  |  |
| RENAL score |  |  |  |  | **0.001** |  |  |  | **0.021** |
| Low (4–6) | **1 (Reference)** | | | |  | **1 (Reference)** | | |  |
| Intermediate (7–9) | **3.088** | | **1.182-8.066** | | **0.021** | **2.249** | **0.714-7.082** | | **0.166** |
| High (10–12) | **12.687** | | **3.259-49.393** | | **<0.001** | **11.490** | **2.022-65.304** | | **0.006** |
| Tumor margin irregularity | **11.333** | | **4.421-29.055** | | **<0.001** | **7.109** | **2.370-21.327** | | **<0.001** |
| Necrosis | **7.242** | | **2.715-19.321** | | **<0.001** | **5.549** | **1.663-18.513** | | **0.005** |
| Pseudocapsule | **0.345** | | **0.153-0.778** | | **0.010** |  |  |  |  |
| Cystic degeneration |  |  |  |  | 0.598 |  |  |  |  |
| ≤25% | 1 (Reference) | | | |  |  |  |  |  |
| 25%-75% | 1.068 | | 0.303-3.761 | | 0.918 |  |  |  |  |
| >75% | 0.321 | | 0.034-2.984 | | 0.317 |  |  |  |  |
| Haemorrhage | 0.906 | | 0.402-2.043 | | 0.812 |  |  |  |  |
| T1 signal intensity |  |  |  |  | 0.089 |  |  |  |  |
| Hypointense | 1 (Reference) | | | |  |  |  |  |  |
| Isointense | 1.556 | | 0.592-4.089 | | 0.370 |  |  |  |  |
| Hyperintense | 0.359 | | 0.079-1.623 | | 0.183 |  |  |  |  |
| T2 signal intensity |  |  |  |  | 0.223 |  |  |  |  |
| Hypointense | 1 (Reference) | | | |  |  |  |  |  |
| Isointense | 1.667 | | 0.147-18.874 | | 0.680 |  |  |  |  |
| Hyperintense | 0.440 | | 0.070-2.764 | | 0.382 |  |  |  |  |
| Microscopic fat |  |  |  |  |  |  |  |  |  |
| **Quantitative MRI parameters** |  |  |  |  |  |  |  |  |  |
| Tumor ADC value (mm2/s) | **0.999** | | **0.998-1.000** | | **0.010** | **0.998** | **0.997-1.000** | | **0.006** |
| HDT-T2 | 0.996 | | 0.992-1.000 | | 0.080 |  |  |  |  |
| Corticomedullary phase |  | |  | |  |  |  |  |  |
| TSICP-CMP | 0.702 | | 0.442-1.113 | | 0.133 |  |  |  |  |
| TCEI-CMP | **0.354** | | **0.155-0.810** | | **0.014** |  |  |  |  |
| Nephrographic phase |  |  |  |  |  |  |  |  |  |
| TSICP-NP | 0.696 | | 0.434-1.115 | | 0.131 |  |  |  |  |
| TCEI-NP | 0.382 | | 0.171-0.852 | | 0.019 |  |  |  |  |
| Excretory phase |  |  |  |  |  |  |  |  |  |
| TSICP-EP | 0.681 | | 0.415-1.117 | | 0.128 |  |  |  |  |
| TCEI-EP | 0.405 | | 0.184-0.890 | | 0.024 |  |  |  |  |
| RCC: renal cell carcinoma, ADC: apparent diffusion coefficient, HDT: heterogeneous degree of tumor, TSICP: signal intensity change percentage of renal tumor, TCEI: tumor-to-cortex enhancement index, CMP: corticomedullary phase, NP: nephrographic phase, EP: excretory phase | | | | | | | | | |

| Supplementary Table 4. The diagnostic performance of different predictive models. | | | | | | | |
| --- | --- | --- | --- | --- | --- | --- | --- |
| Predictive models | AUC (95%CI) | Accuracy | Sensitivity | Specificity | PPV | NPV | F1-score |
| Clinical | 0.706 (0.606-0.806) | 0.676 | 0.756 | 0.617 | 0.596 | 0.771 | 0.667 |
| CEMRI | 0.879 (0.811-0.947) | 0.838 | 0.756 | 0.900 | 0.850 | 0.831 | 0.800 |
| Clinical-CEMRI | 0.907 (0.848-0.965) | 0.857 | 0.778 | 0.917 | 0.875 | 0.846 | 0.824 |
| PPV: positive predictive value, NPV: negative predictive value, AUC: area under the curve. | | | | | | | |
